# *Helicobacter zhangjianzhongii* sp. nov., isolated from dog feces

**DOI:** 10.3389/fgene.2023.1240581

**Published:** 2023-09-26

**Authors:** Hairui Wang, Yixin Gu, Guilan Zhou, Xiaoli Chen, Xin Zhang, Zhujun Shao, Maojun Zhang

**Affiliations:** State Key Laboratory for Infectious Disease Prevention and Control, National Institute for Communicable Disease Control and Prevention, Chinese Center for Disease Control and Prevention, Beijing, China

**Keywords:** Helicobacter zhangjianzhongii, novel species, genomic characteristics, phylogenetic analyses, antimicrobial susceptibility

## Abstract

In 2019, two distinct bacterial isolates were independently isolated from the fecal samples of separate dogs in Beijing, China. These cells exhibit microaerobic, are Gram-negative, motile, and possess a characteristic spiral shape with bipolar single flagellum. They display positive results for the oxidase test while being negative for both catalase and urease. These organisms measure approximately 0.2–0.3 μm in width and 4.5–6 μm in length. The colonies are wet, flat, grey, circular, and smooth with sizes ranging from 1 to 2 mm in diameter after 2 days of growth. However, strains may exhibit variations in size and morphology following extended incubation. Phylogenetic analyses based on the 16S rRNA gene and core genome indicated that these two isolates belong to the genus *Helicobacter* and formed a robust clade that was remains distinctly separate from currently recognized species. These two isolates shared low dDDH relatedness and ANI values with their closest species *Helicobacter canis* CCUG 32756^T^, with these values falling below the commonly cutoff values for strains of the same species. The genomic DNA G + C contents of strain XJK30-2 were 44.93 mol%. Comparing the phenotypic and phylogenetic features between these two isolates and their closely related species, XJK30-2 represents a novel species within the genus *Helicobacter*, for which the name *Helicobacter zhangjianzhongii* sp. nov. (Type strain XJK30-2^T^ = GDMCC 1.3695^T^) is proposed.

## Introduction

The *Helicobacter* genus is classified within the family Helicobacteraceae and the order Campylobacterales. Presently, this genus encompasses 52 officially recognized species alongside 16 species that have yet to receive formal validation (source: https://lpsn.dsmz.de/genus/helicobacter). The *Helicobacter* genus is broadly categorized into gastric (GH) and enterohepatic *Helicobacter* (EHH) species. Members of the *Helicobacter* genus exhibit the following characteristics: they are Gram-negative, spiral, helical, curved, non-spore-forming, or rod-shaped fusiform bacteria, typically measuring between 0.3 and 0.6 μm in width and 1–5 μm in length. In most species, motility is facilitated through the presence of one or multiple flagella, which are encompassed by a protein sheath. Notably, like many campylobacteria, cells have the potential to adopt a coccoid shape in extended cultures or upon exposure to air (On et al., 2017; Ochoa and Collado, 2021; Aydin et al., 2022).

The type species of the genus *Helicobacter* is *Helicobacter pylori*. Convincing evidence has identified *H. pylori* as a causative bacterium of gastric adenocarcinoma, mucosa-associated lymphoid tissue lymphoma, and peptic ulcer in humans (Sonnenberg, 2013). Some non-*H. pylori* species may cause gastritis, typhlocolitis, and hepatitis, mostly in immunocompromised patients and/or as part of multifactorial diseases (Haesebrouck et al., 2009; Mladenova-Hristova et al., 2017; Ochoa and Collado, 2021). While these bacteria naturally inhabit a diverse array of animal species, spanning mammals and birds, their nutritional requirements are quite specific, and they thrive in environments with low levels of oxygen. As a result, there is a dearth of research examining the potential links between diseases and non-H. pylori species. Therefore, there are only a few reports about non-*H. pylori* infection in humans and the mechanisms underlying their ecology and transmission dynamics, host adaptation, and zoonotic potential remain poorly understood (Smet et al., 2018; Ochoa and Collado, 2021). Thus, the pathogenicity and genetic characteristics of non-*H. pylori* remain to be explored.

This study described the taxonomic and genomic characteristics of the novel Helicobacter-like isolates, and the phylogenetic relationships between the isolated strains and their closest relatives were clarified. Based on polyphasic taxonomic analyses, these novel isolates are proposed as novel *Helicobacter* species, designated *Helicobacter zhangjianzhongii* sp. nov. (Type strain XJK30-2^T^ = GDMCC 1.3695^T^).

## Materials and methods

### Sampling, isolation, and culturing

During the investigation of the *Campylobacter* spp. diversity in animals in 2019 in Beijing, isolation was carried out using the *Campylobacter* isolation kit incorporating a membrane filter method (ZC-CAMPY-002, Qingdao Sinova Biotechnology Co., Ltd., Qingdao, China). Briefly, 0.4 mL stool specimen suspension was transferred into 4 mL enrichment buffer which was provided in the kit. The principal component of the enrichment buffer was the modified Preston broth with vancomycin, trimethoprim, and amphotericin B. The enriched suspension was incubated at 37 °C for 24 h in a microaerophilic atmosphere consisting of 5% O_2_, 10% CO_2_, and 85% N_2_. Subsequently, approximately 300 μL of the cultured enrichment suspension was applied as spots onto the surface of filter paper adhered to the double medium plates. These plates consisted of Karmali agar and Columbia agar, each supplemented with 5% defibrinated sheep blood. The medium plates were incubated in a microaerophilic atmosphere at 37 °C for 48 h (Li et al., 2018).

The suspected monoclonal colonies were selected and purified and were subjected to preliminary characterization by PCR amplification and sequencing of 16S rRNA gene (Frank et al., 2008), and subsequently stored at −80 °C in BHI with 20% (v/v) glycerol for further identification.

### Morphological, physiological, and biochemical characteristics

For the assessment of morphological and biochemical traits, cells were cultivated and harvested during the late-exponential growth phase. Gram-staining employed a Gram-staining kit (Baso) (Austrian, 1960) and microscopic observations were carried out using a light microscope (Eclipse Ci-L, NIKON). Morphological features of the type strains of these potential novel species were investigated using transmission electron microscopy. Fresh cells were gently suspended in 0.1 M phosphate-buffered saline (PBS) to attain an OD600 of 1 and then collected via mild centrifugation. The pellet was gently resuspended in a 2% (v/v) glutaraldehyde solution for fixation. Fixation was completed by incubating strains for 1 h on the grid. To enhance visualization, all samples were briefly stained with 2% (w/v) uranyl acetate for 1 min and subsequently subjected to examination using a Hitachi H7700 transmission electron microscope operating at 80 kV.

For biochemical characteristics, the catalase activity was evaluated using a 3% (v/v) H_2_O_2_ solution for bubble production. Further biochemical characteristics were obtained using the identification system of API Campy following the manufacturers’ instructions strictly (bio-Mérieux). Biochemical tests were carried out to characterize the physiology and chemotaxonomy of the isolates. The growth characteristics on Karmali blood agar supplemented with 2% (w/v) NaCl or 3.5% (w/v) NaCl were assessed using established protocols outlined in prior research (On et al., 2017). Following an incubation period of 3–5 days, the growth characteristics of the isolates were examined under various atmospheric conditions. These conditions included anaerobic and aerobic environments at 37°C, as well as microaerobic conditions at 25°C, 37°C, and 42°C. Type strain *H. pylori* ATCC 43504^T^ was used as a control.

### Antimicrobial susceptibility testing

The minimum inhibitory concentrations (MICs) for ten antibiotic classes (macrolides, quinolones, aminoglycosides, chloramphenicol, tetracyclines, ketolides, lincosamides, *β*-lactams, metronidazole, and rifampicin) were determined for isolates using the agar dilution method (ZC-AST-001, Qingdao Sinova Biotechnology Co., Ltd., Qingdao, China) and the gradient strip diffusion method (E-test, bio Mérieux, Nürtingen, Germany) following the manufacturer’s instruction as previously reported (Li et al., 2020; Zhou et al., 2020). The MIC was read as the lowest concentration without visible growth. Type strain *H. pylori* ATCC 43504^T^ was used as a control.

### Genome extraction and sequencing

After culturing, the genomic DNA for sequencing was extracted using the QIAamp DNA Mini Kit (Qiagen, German) according to the manufacturer’s instructions for sequencing. Then the NanoDrop spectrophotometer (Thermo Scientific, Wilmington, DE, USA) was used to measure the concentration and purity of DNAs. The quality requirements were a concentration ≥20 ng/μL and a total amount >2 μg. The purity requirement was as follows: OD260/OD280 value should be between 1.6 and 1.8. The DNA sequencing was performed by an Illumina PE150 platform (Illumina Inc., San Diego, CA, USA) at the Novogene Corporation (Beijing, China) with a depth of 100× coverage. A 350 bp paired-end library was constructed to sequence the genomes, and then 150 bp reads were generated. FastQC (Andrews, 2020) and fastp (Chen et al., 2018) software tools were applied to evaluate and improve the quality of the raw sequence data, respectively. Low-quality reads were removed if the quality scores of ≥3 consecutive bases were ≤ Q30. The clean reads were assembled by SOAPdenovo (Luo et al., 2012).

### Genomic analysis

The assembled sequences were predicted to genes and annotated the function using the Prokka pipeline (Seemann, 2014) and tRNA-scan tool (Lowe and Eddy, 1997). Phage Search Tool (PHAST) web server (Arndt et al., 2016) and phiSpy software (Akhter et al., 2012) were used to search for phage sequences. The antimicrobial resistance genes were predicted using the Comprehensive Antibiotic Resistance Database (CARD) (Alcock et al., 2020). The virulence genes of all the genomes were detected on VFanalyzer (Liu et al., 2019). The dDDH relatedness was calculated and compared using Genome-to-Genome Distance Calculator 3.0 (Meier-Kolthoff et al., 2022). The ANI values were determined by pyani 0.2.10 (Pritchard et al., 2016).

### Phylogenetic and phylogenomic analysis

To determine the phylogenetic positions of strains, 16S rRNA gene PCR amplification was performed with primers 27F and 1492R as previously reported. Each almost-complete sequence of the 16S rRNA gene PCR product was purified, sub-cloned into the pMD18-T vector for 30 min at 16 °C, transformed into *Escherichia coli* DH5α, and the inserted 16S rRNA gene fragment was obtained from a single colony after lysis and sequenced. The newly generated 16S rRNA gene sequences were compared with other *Helicobacter* species by EzBioCloud’s identification service to determine their taxonomic position (Yoon et al., 2017). Multiple sequence alignment of the 16S rRNA gene sequences of the type strains in the genus *Helicobacter* was performed using the MAFFT 7.471 software (Katoh and Standley, 2013) and phylogenetic analysis using the software package MEGA X (Kumar et al., 2018), by the neighbor-joining (NJ) (Saitou and Nei, 1987), maximum-parsimony (MP) (Fitch, 1971) and maximum-likelihood (ML) (Felsenstein, 1981) algorithms with a bootstrap analysis of 1,000 replicates (Felsenstein, 1985) and strain *Arcobacter butzleri* ATCC 49616^T^ was used as an outgroup.

Amino acids from the isolates and other *Helicobacter* species were grouped into clusters using the CD-HIT software (Fu et al., 2012), employing a 40% protein sequence similarity threshold. The representative sequences within each cluster, present across all analyzed genomes, were designated as core sequences. These core sequences were concatenated and aligned using the MAFFT tool. Subsequently, a phylogenomic tree was constructed through FastTree (Price et al., 2009), utilizing the ML algorithm with the JTT substitution model. The visualization of this phylogenomic tree was facilitated by Dendroscope 3.8.3 (Huson and Scornavacca, 2012). The virulence genes and the values of ANI and dDDH were visualized by the pheatmap and ggplot2 packages using R 4.2.2 (Team, 2023), respectively.

### Accession numbers

The GenBank/EMBL/DDBJ accession numbers for the nearly full-length 16S rRNA gene of strains XJK30-2 and CPD2-1 are OP278857 and OP278856, and for the draft genome are JANURN000000000 and JANURO000000000, respectively. In addition to these 2 isolates, genomes from the other type strains of the *Helicobacter* genus were downloaded from NCBI (https://www. ncbi.nlm.nih.gov/).

## Results

### Isolation and phenotypic characterization

**FIGURE. 1:**
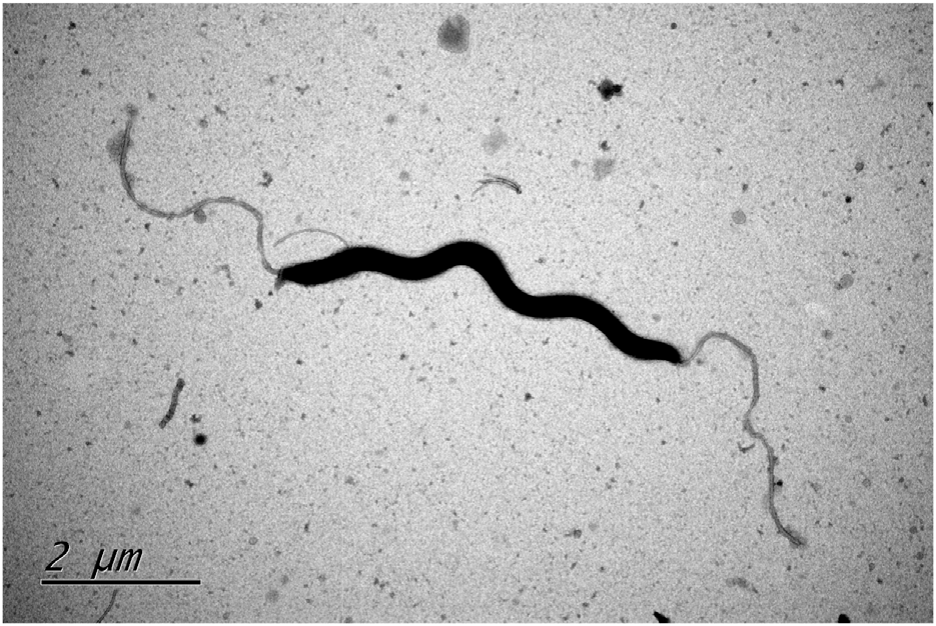
Transmission electron microscope image of type strain *Helicobacter zhangjianzhongii* sp. nov. XJK30-2 from 48 h culture.

In the process, two bacterial isolates designated XJK30-2 and CPD2-1 were independently isolated from the fecal samples of two different dogs. These cells were microaerobic, Gram-negative, motile, and spiral-shaped with bipolar single flagellum, ranging in size of width 0.2–0.3 μm and length 4.5–6 μm (Figure 1). Colonies were circular, 1–2 mm in diameter, smooth, and grey after 48 h of growth on Karmali agar with 5% defibrinated sheep blood. Cells appear coccoid after 5–6 days of incubation or exposure to air. Like *Helicobacter canis*, these two putative isolates are positive for oxidase, gamma-glutamyl transferase, and alkaline phosphatase and negative for catalase and urease activities. They are unable to hydrolyze Hippurate, reduce nitrate, produce hydrogen sulfide, and able to hydrolyze indoxyl acetate. These two isolates were identified initially as *H. canis* according to the results of the sequencing of the 16S rRNA gene. Thus, it was not unexpected that the results of the standard biochemical showed a strong similarity between the composite phenotypic profile observed from the isolates and the phenotypic profile reported previously for *H. canis* CCUG 32756^T^ (Table 1).

**TABLE 1:** Differential characteristics of *Helicobacter zhangjianzhongii* sp. nov. and the type strains of closest related species.

Characteristic	1	2	3	4	5	6
Oxidase	+	+	+	+	+	+
Catalase	-	-	+	-	+	+
Urease	-	-	+	-	-	-
Nitrate reduction	-	-	-	-	-	-
Indoxyl acetate hydrolysis	+	+	(-)	+	+	+
Hippurate hydrolysis	-	-	-	-	-	ND
γ-Glutamyl transpeptidase	+	+	+	+	-	-
Reduction of Triphenyl tetrazolium chloride	+	+	V	+	+	ND
pyrrolidonyl arylamidase	+	+	-	-	-	ND
L-arginine arylamidase	+	+	-	-	V	ND
L-aspartic acid arylamidase	+	+	-	-	-	ND
Alkaline phosphatase	+	+	+	+	+	-
H_2_S	-	-	-	-	-	ND
Grouth at/in/under						
Microaerobic (25°C)	-	-	-	-	-	-
Microaerobic (37°C)	+	+	+	+	+	+
Microaerobic (42°C)	+	+	+	+	(+)	+
Anaerobic (37°C)	-	-	-	-	-	-
Aerobic (37°C)	-	-	-	-	-	-
2% NaCl	-	-	-	-	-	ND
3.5% NaCl	-	-	-	-	-	ND

^1^ *H. zhangjianzhongii* (strain, CPD2-1); 2, *Helicobacter zhangjianzhongii* (strain, XJK30-2); 3, *Helicobacter pylori*; 4, *H. canis*; 5, *H. fennelliae*; 6, *H. macacae*; Data for other species were taken from previous publications (Aydin et al., 2022; Gruntar et al., 2022).

^+^ , 90%–100%; (+), 75%–89%; V, 26%–74%; (−), 11%–25%; -, 0%–10%; ND, not determined.

### Phylogenetic and phylogenomic analysis

**FIGURE. 2:**
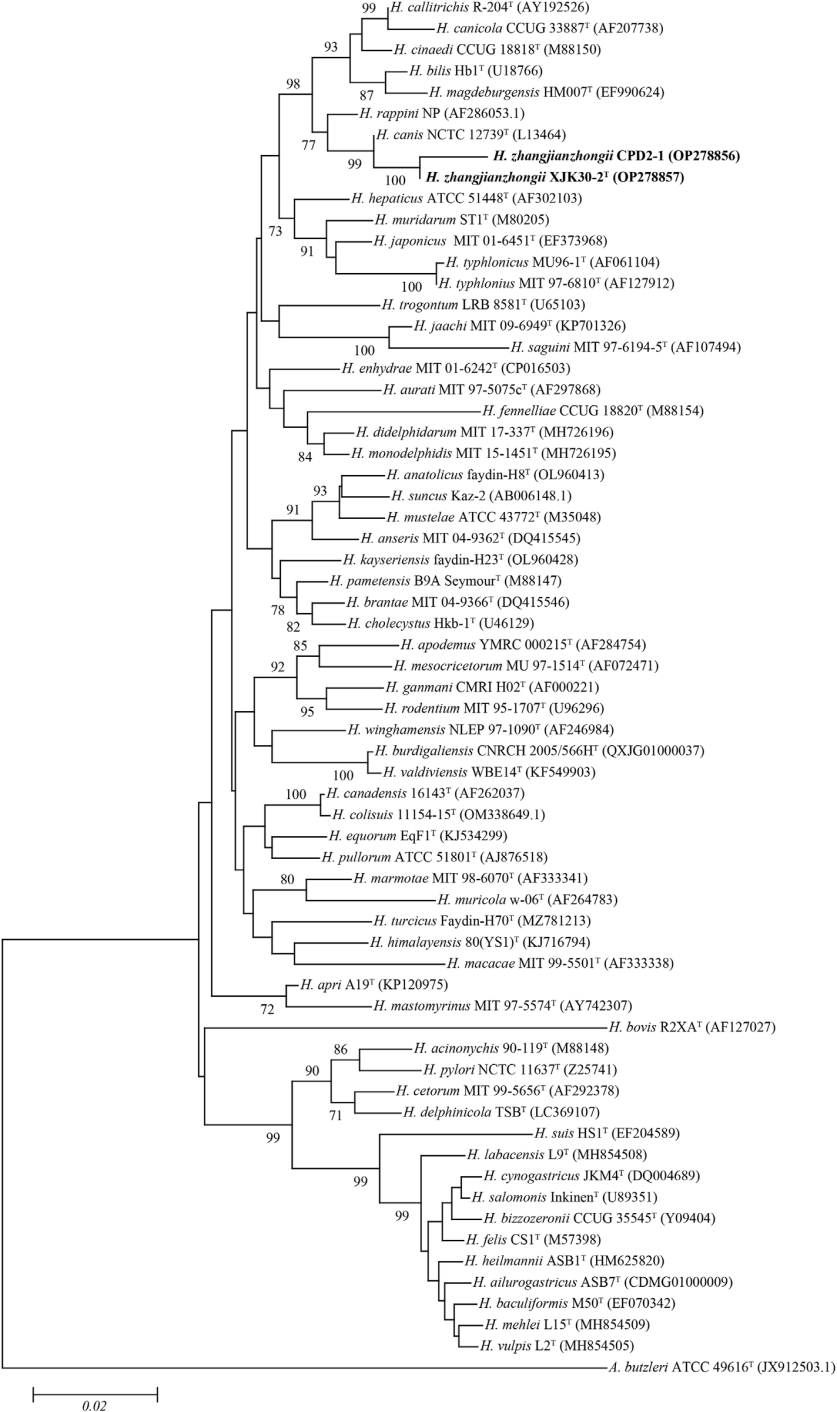
Neighbor-joining phylogenetic tree based on nearly complete 16S rRNA gene showing the relationships between two novel strains and the type strains of the genus *Helicobacter*. Bootstrap values (>70%) based on 1,000 replicates are shown at branch nodes, with *Arcobacter butzleri* ATCC 49616^T^ as an outgroup. Bar, 0.02 changes per nucleotide position. Novel strains are highlighted in bold.

The comparison against the EzTaxon-e database of near full-length 16S rRNA gene sequences revealed that our 2 isolates were most closely related to the representatives of the genus *Helicobacter* (Domain, Bacteria; Phylum, Campylobacterota; Class, Epsilonproteobacteria; Order, Campylobacterales; Family, Helicobacteraceae) (Oren and Garrity, 2021). Isolates XJK30-2 and CPD2-1 were closest to *H. canis* NCTC 12739^T^ (99.35% of 16S rRNA gene identity of strain XJK30-2). The NJ phylogenetic tree (Figure 2) based on the nearly complete 16S rRNA gene sequences revealed that these 2 isolates belong to the genus *Helicobacter* and formed a robust clade that was clearly separate from the currently recognized species, which was supported by the topological results obtained from the ML and MP trees (Supplementary Figure S1; Supplementary Figure S2). Although the identity value between strain XJK30-2 and *H. canis* NCTC 12739^T^ is higher than 98.70% which was the generally accepted threshold for species (Rossi-Tamisier et al., 2015), the results of the phylogenetic tree suggested that these 2 isolates belong to the genus *Helicobacter* and probably represent a novel species.

**FIGURE. 3:**
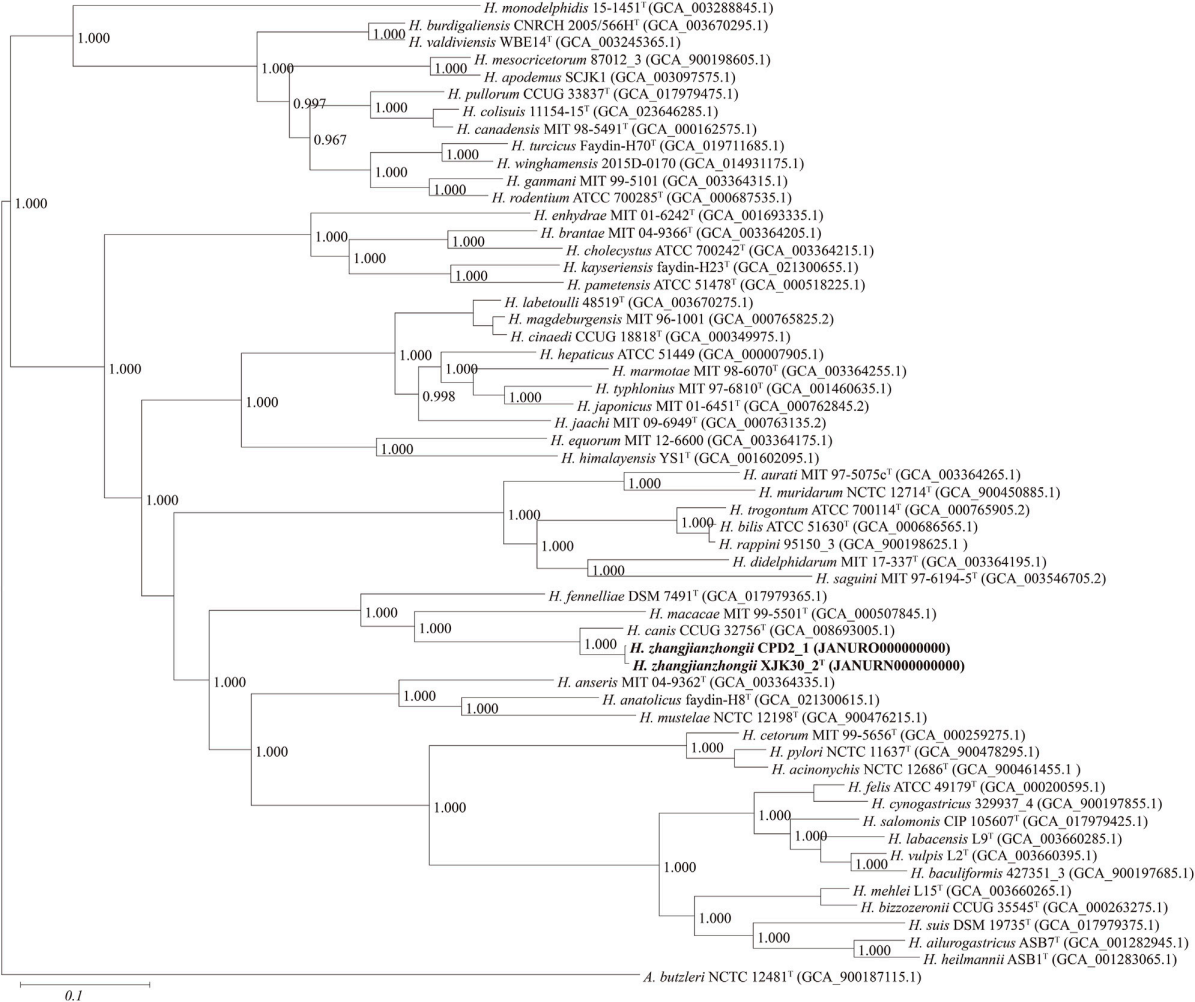
Maximum-likelihood phylogenomic tree based on 173 core genes of the genus *Helicobacter*. Numbers on the tree indicate each split in the tree support values with the Shimodaira-Hasegawa test calculated for 1,000 resamples. The outgroup is *Arcobacter butzleri* ATCC 49616^T^. Novel strains are highlighted in bold.

Based on 40% protein identity, orthologous groups of 173 core genes (Supplementary Table S1) shared by our 2 isolates and all available genomes of the genus *Helicobacter* were extracted and used to build a phylogenomic tree (Figure 3). This phylogenomic tree revealed that strains also formed a robust clade, a result identical to that of phylogenetic trees based on nearly complete 16S rRNA gene sequences, further proving that the isolates belong to the genus *Helicobacter* and probably represent a novel species.

### Genome characteristics

The draft genome of strain XJK30-2 (2.08 Mb) contained 29 contigs which with the longest contig 386 096 bp and the shortest contig 768 bp. N50 and N90 were 249 030 bp and 51 139 bp, respectively. there 1872 coding genes, 2 rRNA, 38 tRNA and 1 CRISPR sequence were predicted by Prokka (Table 2). The genomic DNA G + C content of strain XJK30-2 is 44.93 mol%, which is like the most closely related bacterium, *H. canis* CCUG 32756^T^ (44.97%).

**TABLE 2:** Genomes characteristics of strains *Helicobacter zhangjianzhongii* sp. nov.

Strain	CPD2-1	XJK30-2
Contigs	27	29
Genome size (bp)	2,096 761	2,084 878
Max contig (bp)	386 566	386 096
Min contig (bp)	762	768
N50 (bp)	227 215	249 030
N90 (bp)	49 097	51 139
GC content (mol%)	44.90%	44.93%
CDS	1,894	1,872
rRNA	2	2
CRISPR	1	1
tRNA	38	38

**FIGURE. 4:**
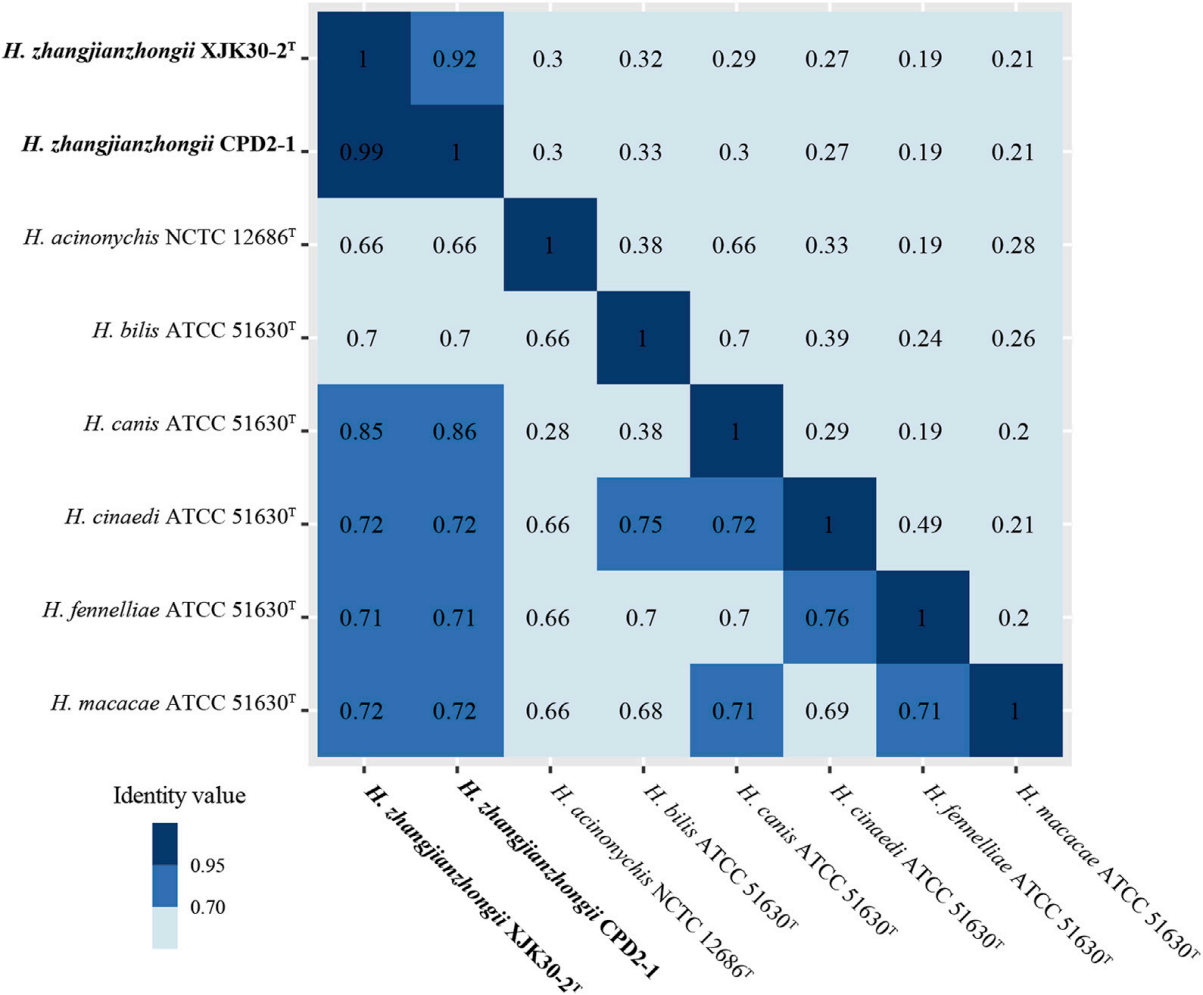
Heatmap of the values ANI (lower diagonal) and dDDH (upper diagonal) among the novel *Helicobacter* strains and other *Helicobacter* species.

The dDDH score within these 2 isolates pair was 91.90% (XJK30-2 and CPD2-1) which were well above 70%, the threshold for species demarcation (Meier-Kolthoff et al., 2022). In contrast, the scores of these 2 strains with the other *Helicobacter* species were below the threshold. Meanwhile, as the gold standard for the delineation of bacterial species (Richter and Rosselló-Móra, 2009), the ANI value within the isolates pair was 99.15% (strains XJK30-2 and CPD2-1), in contrast to below 95%, the cutoff for species demarcation, between our isolates and all established species of *Helicobacter* (Figure 4; Supplementary Table S2).

### Antibiotic resistance and pathogenicity

Antibiotic resistance was demonstrated that strains XJK30-2 and CPD2-1 were resistant to four types of antibiotics, macrolides (erythromycin, azithromycin, and clarithromycin), quinolones (nalidix acid and ciprofloxacin), clindamycin, and rifampicin. In genomes, the prevalence of *rpoB* (I2619V) conferring resistance to fluoroquinolone and rifamycin antibiotics was found among these 2 strains (100.00%). This antibiotic resistance mutation was consistent with the resistance phenotype of resistance to quinolones and rifampicin. however, more antibiotic-resistance genes conferring resistance to macrolides, quinolones, and clindamycin, like mutations in GyrA, GyrB, and the 23S rRNA, were not found in these genomes (Shen et al., 2014; Borges et al., 2015; Gotoh et al., 2018). Further studies should pay more attention to this aspect.

**FIGURE. 5:**
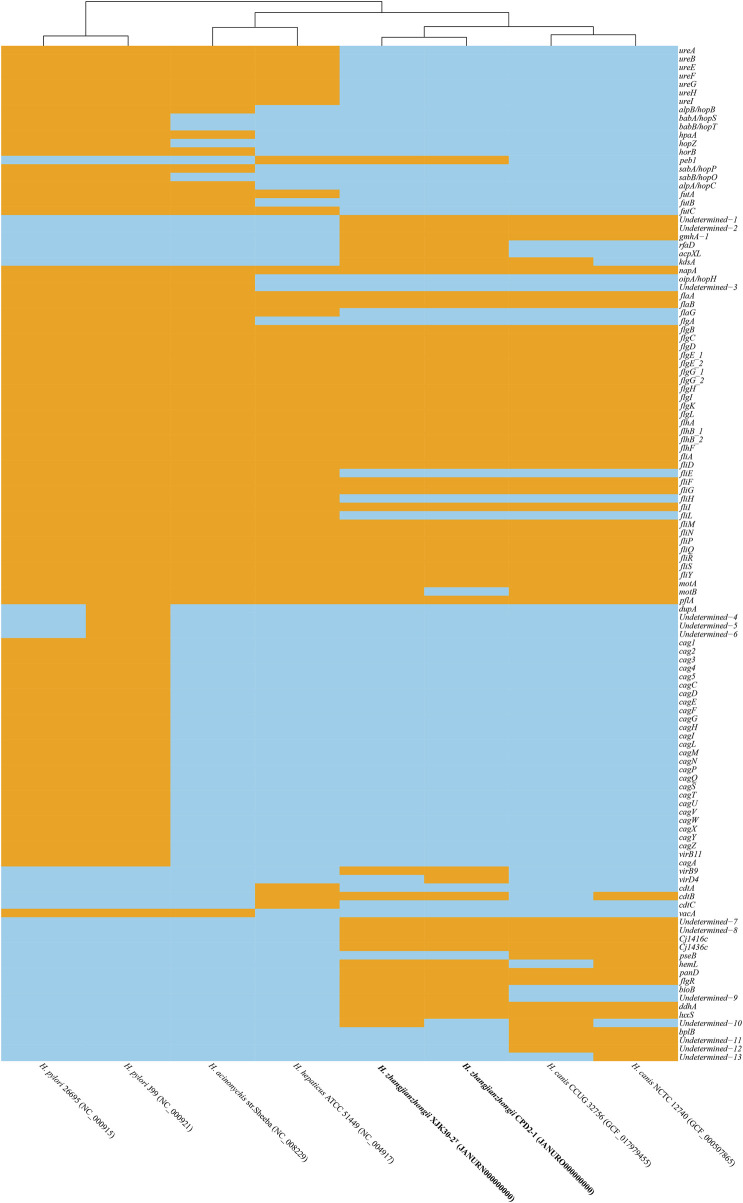
Heatmap of the distribution of virulence genes. Orange indicates the presence of the virulence genes, and skyblue indicates the absence of the virulence genes.

Within the genomes of these strains, a multitude of *Helicobacter* virulence-associated genes have been identified. These encompass genes potentially associated with acid resistance, adherence, immune evasion, immune modulation, and motility, among others. Notably, the virulence-linked components of urease, cag PAI (pathogenicity island), and the entire cytolethal distending toxin were conspicuously absent from both genomes. The primary virulence-associated attributes were centered around immune evasion and motility. A comprehensive depiction of the virulence genes can be found in Figure 5, providing a detailed insight into their distribution and relevance.

## Discussion

Most reported *Helicobacter* infections were caused by *H. pylori*. Convincing evidence has identified *H. pylori* as a causative bacterium of multiple stomach diseases in humans (Sonnenberg, 2013). However, increasing recognition of other emerging *Helicobacter* pathogens has been recognized as important pathogens in humans and animals (Haesebrouck et al., 2009; Mladenova-Hristova et al., 2017; Ochoa and Collado, 2021). Therefore, the mechanisms underlying their ecology and transmission dynamics, host adaptation, and zoonotic potential of non-*H. pylori* remain to be explored (Smet et al., 2018; Ochoa and Collado, 2021).

*Helicobacter zhangjianzhongii* sp. nov. has typical phenotypic and genomic characteristics of *Helicobacter*. Further, this species could be distinguished from its closest related species *H. canis* by their positive for pyrrolidonyl arylamidase, L-arginine arylamidase, and L-aspartate arylamidase. Although the identity value of the nearly complete 16S rRNA gene sequences between novel species and closest related species is higher than threshold for species (Rossi-Tamisier et al., 2015), the results of the phylogenetic and phylogenomic analysis suggested that this species belong to the genus *Helicobacter* and probably represent a novel species. The results of dDDH score and ANI value affirmed that these two isolates represented a novel species of the genus *Helicobacter*.

Antimicrobial resistance in *Helicobacter* is a pressing concern, complicating the treatment of infections caused by this bacterium. In the previous reports, resistance to macrolides has been reported in *H. pylori*, *Helicobacter cinaedi, Helicobacter fennelliae*, and *Helicobacter pullorum* (Rimbara et al., 2013; Borges et al., 2015; Gotoh et al., 2018; Ruksasiri et al., 2018; Nukui et al., 2020) and to quinolones in *H. pylori, Helicobacter canadensis*, *H. cinaedi*, *H. fennelliae* and *H. pullorum* (Ceelen et al., 2005; Zanoni et al., 2011; Hassan et al., 2014; Gotoh et al., 2018; Ruksasiri et al., 2018; Nukui et al., 2020). Between 2012 and 2015, *H. pylori* resistance to clindamycin was 8.3%–100% (Contreras-Omaña et al., 2021). Resistance to rifampin also has been found in *H. pylori*, *H. canadensis*, *H. pullorum*, and “*Helicobacter winghamensis*” (Shen et al., 2014). The prevalence of resistance to commonly used antibiotics posing a significant threat to public health. As such, continued monitoring and surveillance of *Helicobacter* antimicrobial resistance patterns is crucial to inform effective treatment strategies and to curb the spread of resistance.

## Conclusion

A polyphasic approach, including DNA sequencing and analysis (16S rRNA and whole genome sequencing), electron microscopy, and a wide range of biochemical tests, as suggested by On et al. (On et al., 2017), provided sufficient evidence to distinguish these 2 isolates from their closest related type strains and to confirm that they represent a novel species. With XJK30-2 as the type strain, we suggest the names *Helicobacter zhangjianzhongii* sp. nov. for the novel member of the genus *Helicobacter*.

## Description of *Helicobacter zhangjianzhongii* sp. nov.

*Helicobacter zhangjianzhongii* (zhang.jian.zhong’i.i, N. L. gen. n. *zhangjianzhongii* of Jianzhong Zhang, a microbiologist in China CDC known for his contribution to the control and prevention of infectious diseases in China, especially in the diagnosis and prevention of *H. pylori*).

The cells exhibit characteristics of being Gram-negative, motile, and spiral-shaped, possessing a single bipolar flagellum. Following 48 h of growth on Karmali or Columbia blood agar under a microaerophilic environment at 37°C, their dimensions span between 0.2 and 0.3 μm in width and 4.5–6 μm in length. Colonies produced by these cells showcase a wet, flat, circular, and smooth appearance, with diameters ranging from 1 to 2 mm after a 2-day incubation period. Following an extended incubation period, they might display variability in both size and morphology. Notably, no hemolysis is observed on blood agar. Upon immediate examination, the cells exhibit motility, facilitated by their long bipolar single flagella.

Both of these strains exhibit positive results for oxidase, gamma-glutamyl transferase, alkaline phosphatase, pyrrolidonyl arylamidase, L-arginine arylamidase, and L-aspartate arylamidase, while demonstrating negative outcomes for catalase and urease activities. They do not possess the ability to hydrolyze Hippurate or reduce nitrate, nor can they generate hydrogen sulfide. However, they do exhibit the capacity to hydrolyze indoxyl acetate. In terms of antibiotic sensitivity, both strains showcase sensitivity to gentamicin, streptomycin, chloramphenicol, florfenicol, tetracycline, and telithromycin. Conversely, they exhibit resistance to erythromycin, azithromycin, clarithromycin, nalidixic acid, ciprofloxacin, clindamycin, and rifampicin.

The type strain XJK30-2^T^ (=GDMCC 1.3695^T^), isolated from the dogs’ feces in 2019 in Beijing, has a DNA G + C content of 44.93 mol%.

## Data Availability

The datasets presented in this study can be found in online repositories. The names of the repository/repositories and accession number(s) can be found in the article/Supplementary Material.

## Supplementary material

Supplementary Figure S1 Maximum-likelihood phylogenetic tree based on nearly complete 16S rRNA gene showing the relationships between two novel strains and the type strains of the genus Helicobacter. Bootstrap values (>70%) based on 1,000 replicates are shown at branch nodes, with Arcobacter butzleri ATCC 49616T as an outgroup. Novel strains are highlighted in bold.

Supplementary Figure S2 Maximum-parsimony phylogenetic tree based on nearly complete 16S rRNA gene showing the relationships between two novel strains and the type strains of the genus Helicobacter. Bootstrap values (>70%) based on 1,000 replicates are shown at branch nodes, with Arcobacter butzleri ATCC 49616T as an outgroup. Novel strains are highlighted in bold.

Supplementary Table S1 Core genes extracted from the clusters grouped by CD-HIT.

Supplementary Table S2 ANI (lower diagonal) and dDDH (upper diagonal) among the novel Helicobacter strains and other Helicobacter species.

## Abbreviations

GH, gastric *Helicobacter*; EHH, enterohepatic *Helicobacter*; ANI, average nucleotide identity; dDDH, digital DNA-DNA hybridization; ML, maximum likelihood; MP, maximum parsimony; NJ, neighbor-joining; ME, minimum evolution.
