# Supplementary figures and images for: Helicobacter zhangjianzhongii sp. nov., isolated from dog feces
Source: Front Genet. 2023 Sep 26;14:1240581. doi: 10.3389/fgene.2023.1240581 (PMC10562538; doi:10.3389/fgene.2023.1240581)

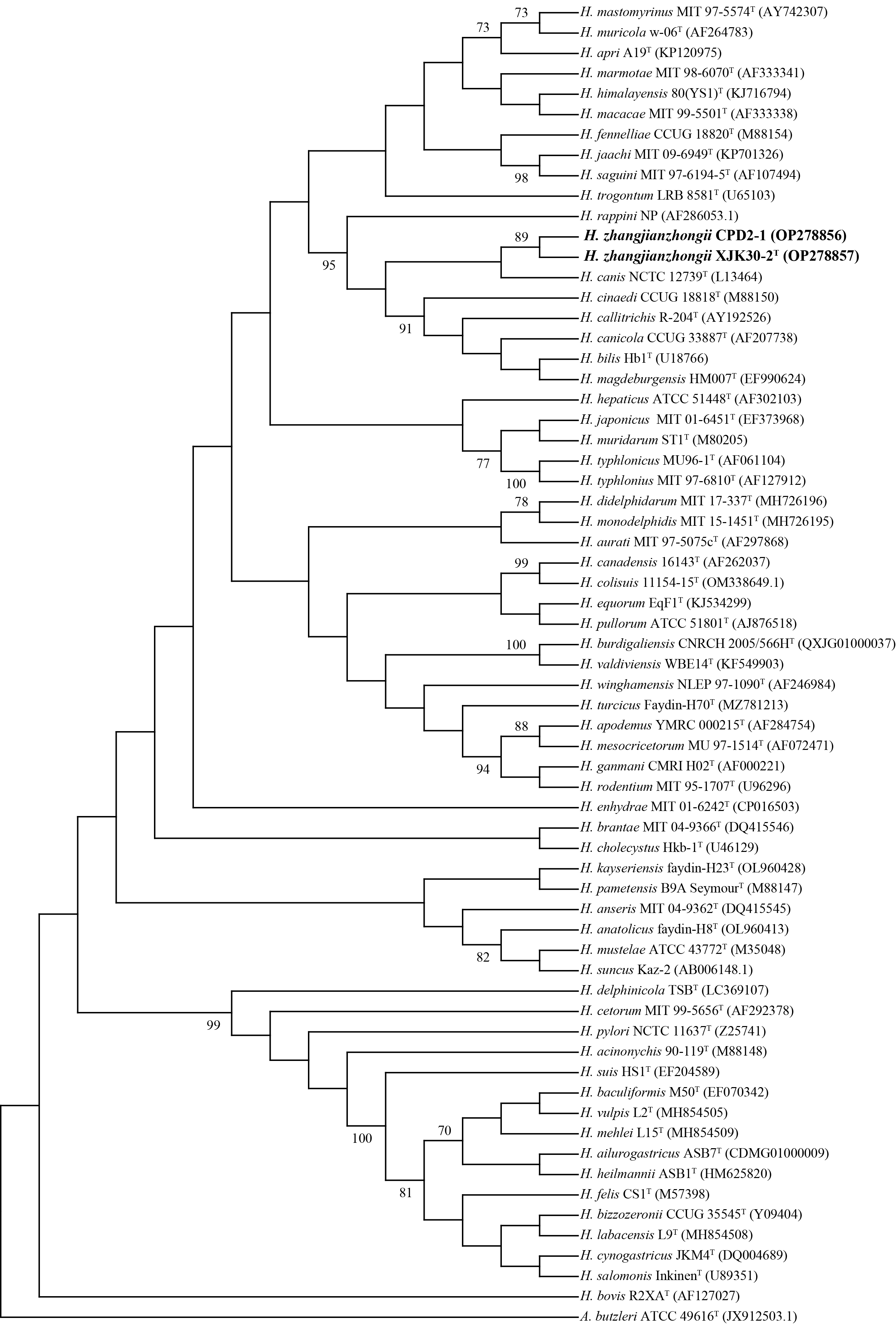

Supplement: Supplementary file 2 [file Image2.TIF]

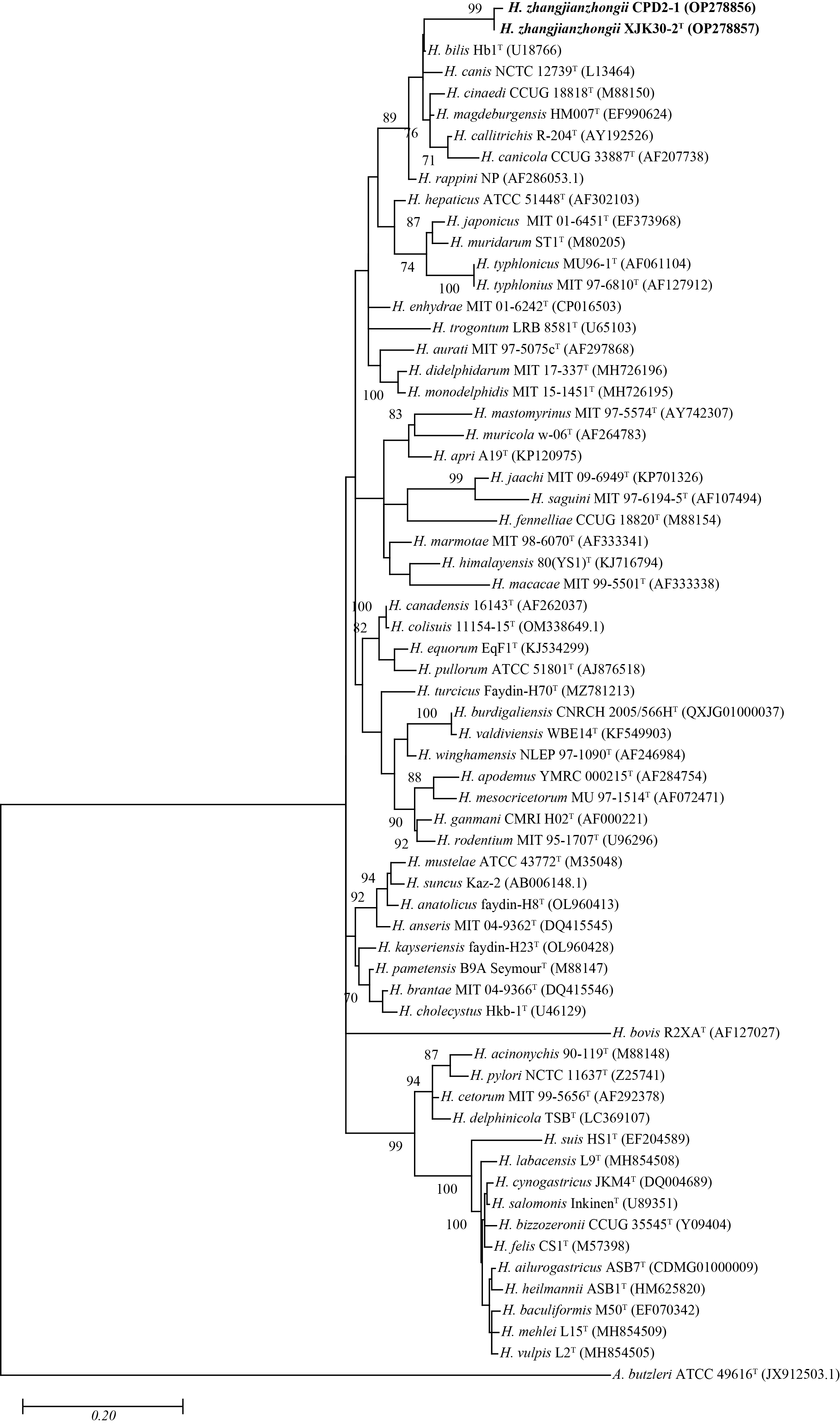

Supplement: Supplementary file 3 [file Image1.TIF]
